# Risk factors for surgical site infections following open versus laparoscopic colectomies: a cohort study

**DOI:** 10.1186/s12893-021-01379-w

**Published:** 2021-10-25

**Authors:** Tomer Hoffman, Pnina Shitrit, Michal Chowers

**Affiliations:** 1 Infectious Diseases Unit, Meir medical Center, Kfar-Saba, Israel; 2 Infection Control Unit, Meir medical Center, Kfar-Saba, Israel; 3 Sackler Medical School, Tel-Aviv University, Tel-Aviv, Israel

**Keywords:** Colorectal surgery, Laparoscopic, Surgical site infection, Chronic obstructive pulmonary disease, Risk factors

## Abstract

**Background:**

Surgical site infections (SSIs) are among the most common healthcare-associated infections. Evaluating risk factors for SSIs among patients undergoing laparoscopic and open colorectal resections can aid in selecting appropriate candidates for each modality.

**Methods:**

A cohort of all consecutive patients undergoing elective colorectal resections during 2008–2017 in a single center was analyzed. SSIs were prospectively assessed by infection control personnel. Patient data were collected from electronic medical records. Risk factors for SSIs were compared between patients who underwent laparoscopic and open surgeries. A multivariate analysis was performed for significant variables.

**Results:**

During the study period, 865 patients underwent elective colorectal resection: 596 laparoscopic and 269 open surgeries. Mean age was 68.2 ± 15.1 years, weight 72.5 ± 18.3 kg and 441 (51%) were men. The most common indication for surgery was malignancy, in 767 patients (88.7%) with inflammatory bowel diseases and diverticulitis following (4.5% and 3.9%, respectively). Patients undergoing laparoscopic surgery were younger, had fewer comorbidities, shorter pre-operative hospitalizations, lower risk index scores, and lower rates of SSI, compared with open surgery. Independent risk factors for SSI following laparoscopic surgery were chronic obstructive pulmonary disease [odds ratio (OR) 2.655 95% CI (1.267, 5.565)], risk index ≥ 2 [OR 2.079, 95% CI (1.041,4.153)] and conversion of laparoscopic to open surgery [OR 2.056 95%CI (1.212, 3.486)]. Independent risk factors for SSI following open surgery were immunosuppression [OR 3.378 95% CI (1.071, 10.655)], chronic kidney disease [OR 2.643 95% CI (1.008, 6.933)], and need for a second dose of prophylactic antibiotics [OR 2.519 95%CI (1.074, 5.905)].

**Conclusions:**

Risk factors for SSIs differ between laparoscopic and open colorectal resections. Knowledge of specific risk factors may inform patient selection for these modalities.

## Background

Surgical site infections (SSIs) are among the most common healthcare-associated infections (HAIs), accounting for approximately 20% of HAIs in the United States [1] and Europe [2]. Colorectal surgery is associated with a particularly high risk of SSI, with SSI rates ranging from 9 to 27% [3, 4].

A Cochrane review found that antibiotic prophylaxis reduced the risk of SSI following colorectal surgery from 39 to 13% [5].

The causative organisms of SSIs after colorectal surgery originate in the intestinal lumen, and are primarily *Enterobacterales* and anaerobic bacteria.

The risk for an SSI following colorectal surgery relates both to patient characteristics and to procedure-related factors. Superficial SSIs, deep SSIs and organ-space infections differ in their specific risk factors for SSIs [6].

Laparoscopy is an increasingly popular surgical approach for colorectal surgery in general and for colorectal resections in particular, as it is associated with improved post-operative morbidity, lower SSI rates and reduced post-operative mortality rates [7].

Risk factors for SSIs following laparoscopic colorectal resections are not as well described as for an open surgical approach, due to limitations of previous studies, including heterogeneous patient populations and surgical indications, limited sample sizes and inconsistent results [8–10]. In addition, data comparing risk factors for SSIs following laparoscopic and open colorectal resections in similar patient populations are lacking.

The primary objective of this cohort study was to compare the risk factors for SSIs between patients undergoing laparoscopic vs. open colorectal resections.

## Methods

### Study design and patients

This study was based on a prospective cohort consisting of all consecutive patients age 18 years or older who underwent elective (non-urgent) colorectal resection surgery at Meir Medical Center in Israel from January 1, 2008 through December 31, 2017.

### Data collection

During the study period, SSIs were prospectively monitored by dedicated infection control practitioners (ICP) as part of an ongoing surveillance program of post-operative colorectal patients. The surveillance included direct observation of the patient and the surgical site 72 h post-surgery, a phone call to patients at day 30 after surgery and assessment of patient electronic medical records, including outpatient clinic visits, and antibiotic prescription in the community.

Definition of SSIs and their classification (superficial wound infection, deep wound infection or organ-space infection) were based on CDC guidelines [11]. Thus anastomotic leaks were considered space-organ infections and were not included as a cause for SSIs.

Standardized data collection was prospectively performed throughout the surveillance period. Additional data were historically collected from electronic medical records. Data on the following parameters was collected: age, sex, comorbidities, previous abdominal surgeries, risk index [12], indication for surgery, colonic segment resected, duration of pre-operative hospitalization, surgery duration, prophylactic antibiotics, ileostomy/colostomy, conversion of laparoscopic to open surgery, SSI occurrence, microbial pathogens in wound/surgical/drain/blood sample cultures, antibiotic therapy, re-operation, hospitalization duration, re-admission and death within 30 days of surgery.

### Statistical analysis

Descriptive data of continuous and ordinal variables are presented as mean, median and standard deviation, Categorical variables are presented as percentages.

Chi-square, independent t-test and Wilcoxon rank-sum tests were used to test for differences in characteristics between patient groups. Logistic regression models were used to test the association between SSI and each of the variables in the laparoscopic and open surgery groups. Variables deemed important or variables with P-values ≤ 0.1 were eligible for multivariate regression analysis. Odds ratio values are presented, along with 95% confidence intervals (CI). Statistical significance was defined as P < 0.05. All data were analyzed using SPSS software, version 25.0 (IBM Corp. Armonk, NY, USA).

### Ethical considerations

The study was approved by the Institutional Review Board of Meir Medical Center (59-17 MMC). Due to the retrospective nature of the study the need for consent was waived.

## Results

Over the 10-year study period, 865 patients underwent elective colorectal resections, 596 laparoscopic surgery and 269 open surgery. The mean age was 68.2 ± 15.1 years, the mean weight was 72.5 ± 18.3 kg and 441 (51%) were men. The most common indication for surgery was malignancy, in 767 patients (88.7%), with inflammatory bowel diseases and diverticulitis following (4.5% and 3.9%, respectively). A total of 319 patients (36.9%) had undergone abdominal surgery prior to the index operation. SSIs were diagnosed in 197 patients (22.8%), of which 109 (12.6%) were superficial and 26 (3%) were deep wound infections and 59 (6.8%) were organ/space infections. The most common bacteria identified was *Escherichia coli* in 75 cultures, *Enterococcus* spp. in 29 cultures, *Pseudomonas* spp. in 27 cultures and *Staphylococcus aureus* in 7. Re-operation was performed in 52 (6%) patients and 17 patients (2%) died within 30 days of surgery.

The characteristics of the patients undergoing laparoscopic versus open surgery differed in several aspects (Table 1). They were younger, had fewer comorbidities, had shorter hospital stays prior to surgery, had a lower risk index scores and, as expected, fewer SSIs.

**Table 1:** Characteristics of patients undergoing laparoscopic versus open surgery

Characteristic	Laparoscopic N = 596 (%)	Open N = 269 (%)	P-value
Age, years	67.02 ± 15.96	70.82 ± 15.50	0.001
Men, N (%)	291 (48.8)	150 (55.8)	0.059
Weight, kg	73.61 (± 18.80)	70.22 (± 16.99)	0.012
DM	137 (23)	77 (28.6)	0.075
CVD	124 (20.8)	71 (26.4)	0.069
CKD	37 (6.2)	22 (8.2)	0.287
COPD	38 (6.4)	28 (10.4)	0.039
Immuno-suppression	29 (4.9)	15 (5.6)	0.660
Malignancy	456 (76.5)	218 (81.0)	0.137
IBD	28 (4.7)	22 (8.2)	0.004
Prior surgery	198 (33.2)	121 (45)	0.001
Pre-op hospital days	1.6 ± 2.33	2.52 ± 4.23	< 0.001
Rectal surg	83 (13.9)	52 (19.3)	0.043
Risk Index			0.008
0	173	81	
1	328	124	
≥ 2	93	64	
Surgery duration > 75%	331 (56.1)	103 (38.6)	< 0.001
Stoma created	49 (8.2)	42 (15.6)	0.001
Appropriate prophylaxis	336 (67.7)	114 (47.7)	< 0.001
2^nd^ antibiotic dose	62 (10.4)	28 (10.4)	0.998
SSI	110 (18.5)	87 (32.3)	< 0.001
hospital days	7.94 ± 9.09	13.99 ± 25.27	< 0.001
Reoperation	23 (3.9)	29 (10.8)	< 0.001
Readmission	69 (11.6)	49 (18.2)	0.008
Mortality	7 (1.2)	10 (3.7)	0.013

*DM* diabetes mellitus; *CVD* cardiovascular disease; *CKD* chronic kidney disease, *COPD* congestive obstructive pulmonary disease; *IBD* irritable bowel disease; *SSI* surgical site infection

We investigated risk factors for SSIs for each surgical modality (Table 2).

**Table 2:** Risk-factors for surgical site infection (SSI) with laparoscopic and open surgeries

Risk-factor	Laparoscopic (596)			Open (269)		
	SSI N = 110 (%)	No SSI N = 486(%)	P-value	SSI N = 87 (%)	No SSI N = 182 (%)	P-value
Age, years	68.1 ± 14.6	66.8 ± 15.5	0.414	70.7 ± 14.1	70.9 ± 14.8	0.924
Men, N	60 (54.5)	231 (47.5)	0.184	55 (63.2)	96 (52.2)	0.089
Weight, kg	76.5 ± 23.1	73.0 ± 17.6	0.133	72.4 ± 17.8	69.2 ± 16.6	0.149
DM	33 (30)	104 (21.4)	0.053	21 (24.1)	56 (30.8)	0.26
CVD	25 (22.7)	99 (20.4)	0.582	29 (33.3)	42 (23.1)	0.074
CKD	12 (10.9)	25 (5.1)	0.024	12 (13.8)	10 (5.5%)	0.02
COPD	14 (12.7)	24 (4.9)	0.003	13 (14.9)	15 (8.2)	0.092
Immunosuppression	8 (7.3)	21 (4.3)	0.194	8 (9.2)	7 (3.8)	0.074
Malignancy	84 (76.4)	372 (76.5)	0.968	66 (75.9)	152 (83.5)	0.134
IBD	7 (6.4)	21 (4.3)	0.153	9 (10.3)	13 (7.1)	0.37
Prior surgery	34 (30.9)	164 (33.7)	0.569	41 (47.1)	80 (44)	0.625
Pre-op hospital days	1.9 ± 2.9	1.5 ± 2.2	0.253	2.9 ± 5.2	2.4 ± 4.0	0.40
Rectal surg	16 (14.5)	67 (13.8)	0.835	23 (26.4)	29 (15.9)	0.041
Risk Index			0.005			0.082
0	20	155		23	58	
1	65	263		36	88	
≥ 2	25	68		28	36	
Surgery duration > 75%	59 (63.9)	262 (54.4)	0.071	40 (46.5)	63 (34.8)	0.066
Stoma created	13 (11.8)	36 (7.4)	0.128	17 (19.5)	25 (13.7)	0.220
Converted to open	27 (24.5)	63 (13)	0.002			
Appropriate prophylaxis	86 (81.1)	366 (76.3)	0.279	58 (67.4)	99 (57.6)	0.125
Second antibiotic dose	18 (16.4)	44 (9.1)	0.023	15 (17.2)	13 (7.1)	0.011
Post-op hospital days	13 ± 18.9	6.7 ± 3.6	< 0.001	20.0 ± 23.2	11.1 ± 25.8	0.005
Reoperation	17 (15.5)	6 (1.2)	< 0.001	23 (26.4)	6 (3.3)	< 0.001
Readmission	34 (30.9)	35 (7.2)	< 0.001	21 (24.1)	28 (15.4)	0.082
Mortality	2 (1.8)	5 (1)	0.488	8 (9.2)	2 (1.1)	0.002

*DM* diabetes mellitus; *CVD* cardiovascular disease; *CKD* chronic kidney disease, *COPD* congestive obstructive pulmonary disease; *IBD* irritable bowel disease

Risk factors for SSIs following laparoscopic surgeries were chronic kidney disease (CKD), chronic obstructive pulmonary disease (COPD), and diabetes mellitus (DM), high risk index score, need for a second intra-operative prophylactic antibiotic dose, and conversion of laparoscopic surgery to open surgery. We also included age, sex and weight in the regression analysis. Independent risk factors for infection following laparoscopic surgeries found in multivariate regression analysis were: COPD, OR 2.655, 95% CI (1.267, 5.565); Risk index ≥ 2, OR 2.079, 95% CI (1.041,4.153); and conversion to open surgery, OR 2.056, 95%CI (1.212, 3.486).

Risk factors for SSI following open surgeries were CKD, rectal surgery, and the need for a second intra-operative prophylactic antibiotic dose. The regression model included age, sex, weight, immunosuppression, cardiovascular disease (CVD), COPD, and risk index. Independent risk factors for SSIs following open surgeries found in multivariate regression analysis were: immunosuppression, OR 3.378, 95% CI (1.071, 10.655); CKD, OR 2.643 95% CI (1.008, 6.933); and the need for a second intra-operative prophylactic antibiotic dose, OR 2.519, 95% CI (1.074, 5.905).

## Discussion

In this study of 865 patients undergoing elective colorectal surgery, we found different risk factors for SSIs among patients undergoing laparoscopic vs. open colorectal resections. Focusing on basic patient characteristics, COPD was a risk factor for an SSI following laparoscopic surgery but not following open surgery, whereas CKD and immunosuppression were risk factors following open surgery only. Many studies focused on modifiable risk factors, such as prophylactic antibiotics, surgical techniques, and bowel preparation. Patient characteristics are not subject to change but optimal patient selection for each surgical modality may affect the risk of infection.

Laparoscopic colorectal resection has become increasingly popular in the field of colorectal surgery, increasing in incidence from 15% of all colorectal resections in 2007 [13] to 56% in a study published in 2019. This rise in popularity is in part related to lower complication rates, including lower rates of SSIs, seen in laparoscopic surgery [14].

Laparoscopic surgery has been found safe and superior to open surgery in numerous patient outcomes and in a variety of patient populations. In our study, laparoscopic surgery was selected for most patients (69%). The research question we sought to answer was whether there were specific patient populations for which open surgery would be preferable with regard to the risk of SSI. Chronic obstructive pulmonary disease was found to be an independent risk factor for SSIs following laparoscopic but not open colorectal resection. Laparoscopic surgery, although minimally invasive, requires the creation of a pneumoperitoneum, thus reducing important pulmonary function parameters, such as dynamic compliance and functional residual capacity, which increases the risk of hypoxemia [15].

Laparoscopic surgery might increase the risk for an SSI among patients with COPD. In a study by Liao et al. patient outcomes following laparoscopic cholecystectomies were worse in 3954 COPD patients compared to age-and gender-matched non-COPD patients. They found increased rates of intensive care unit admission, longer durations of mechanical ventilation and hospitalization, and higher mortality rates. SSIs were not assessed in that study [16]. Other studies identified COPD as risk factor for SSIs in a mixed patient population undergoing colorectal surgery [17, 18], but the contribution of this risk factor for infection in the different surgical modalities could not be assessed. Drosdeck et al. did not find COPD to be a risk factor for SSIs in a study of 400 patients undergoing laparoscopic colorectal surgery. Of note, inclusion criteria in this study were different, and all patients whose surgeries were converted to open surgeries were excluded [19]. A large study of 4397 COPD patients compared complications of laparoscopic versus open colorectal surgery. In that study, laparoscopic surgery improved most surgical outcomes including ventilator requirement and SSIs by almost 50%. Of note, preoperative steroid use was more frequent in the open surgery cohort, as well as partially-dependent functional status, perhaps favoring the laparoscopic surgery group in relation to the risk of SSI [20]. An important factor in reconciling our results with those of Sujatha-Bhaskar et al. [20] is that the COPD grade and severity were not assessed in either study, possibly resulting in inclusion of significantly heterogeneous COPD patient populations, making comparison more difficult. A study with a more accurate assessment of COPD severity is needed before any conclusions can be drawn.

In our study, patients who had open surgery were older, had more comorbidities and were more likely to have undergone prior abdominal surgeries. We observed a more complex clinical course in patients undergoing open surgeries, with longer hospital stays and increased re-admission, re-operation and mortality rates. Immunosuppression and CKD were independent risk factors for SSIs following open but not laparoscopic surgery. Recovery time and hospital stays are shorter after laparoscopic surgeries, an effect found in patients with malignancies as well [13]. With regard to the risk of infection, immunosuppressed patients might benefit most from shorter hospital stays, which are observed more often following laparoscopic surgery.

Advanced age per se should not be considered an indication for open surgery. In a meta-analysis comparing laparoscopic and open surgeries in an elderly population, overall morbidity, as well as cardiovascular complications were lower after laparoscopic surgery [21, 22].

Other independent risk factors for SSIs included conversion of laparoscopic surgery to open surgery and the need for a second intra-operative prophylactic antibiotic dose in open surgeries; both are markers of a more complicated surgical course. Although this information is only available post-operatively, it may still serve as an indication for increased vigilance in search of infection among post-operative patients.

This study had several limitations. It was a single center study and some of the data were collected retrospectively from patient records. Body mass index, a known risk factor for SSIs, was missing from many records, forcing us to use weight as a surrogate marker. Of note, SSI, our major outcome, was assessed prospectively in real-time by experienced ICP.

## Conclusion

Patient characteristics are known, non-modifiable risk factors for SSIs. We suggest that risk factors for SSIs differ between laparoscopic and open colorectal resections, and that knowledge of specific risk factors for SSIs may inform selection of the appropriate surgical modality for every patient.

## Data Availability

Data will be available per request (chowersm@post.tau.ac.il).

## Abbreviations

SSIs: Surgical site infections
OR: Odds ratio
ICP: Infection control practitioners
CKD: Chronic kidney disease
COPD: Chronic obstructive pulmonary disease
DM: Diabetes mellitus
CVD: Cardiovascular disease
